# HPV-Associated Gene Signatures in Bladder Cancer: A Comprehensive Prognostic Model and its Implications in Immunotherapy

**DOI:** 10.7150/ijms.98334

**Published:** 2025-01-01

**Authors:** Zhicheng Tang, Yuxin Qian, Ni Wang, Yinqiu Chen, Haojun Huang, Jiahao Zhang, Hongcheng Luo, Zechao Lu, Zhibiao Li, Zhaohui He, Fucai Tang

**Affiliations:** 1 Department of Urology, The Eighth Affiliated Hospital, Sun Yat-sen University, Shenzhen, Guangdong 518033, China.; 2 School of Medicine, Sun Yat-sen University, Shenzhen 518107, China.

**Keywords:** Bladder cancer, Prognostic model, Human papillomavirus (HPV)

## Abstract

**Background:** Evidence increasingly indicates that HPV infection plays a pivotal role in the initiation and progression of bladder cancer (BC). Yet, determining the predictive value of HPV-associated genes in BC remains challenging.

**Methods:** We identified differentially expressed HPV-associated genes of BC patients from the TCGA and GEO databases. We screened prognostic genes using COX and LASSO regression, subsequently establishing a risk prediction model. The model's precision and clinical relevance were gauged using Kaplan-Meier survival analyses and ROC curves. Functional enrichment, immune cell infiltration, and drug sensitivity analyses were performed across both high-risk and low-risk sets. PCR assays were utilized to measure the expression levels of genes.

**Results:** We identified 13 HPV-associated genes for our risk model. Among these, FLRT2, HOXC5, LDLR, SCD, GRM7, DSC1, EMP1, and HMGA1 were identified as risk contributors, while LPA, SERPINA6, ZNF124, ETV7, and SCO2 were deemed protective. Cox regression analysis verified that our model provides an independent prediction of overall survival (OS) in bladder cancer (BC) patients. Gene Ontology (GO) analysis revealed predominant gene enrichment in wound healing, extracellular matrix composition, and collagen-rich extracellular matrices. KEGG pathway analysis highlighted primary enrichment areas, including focal adhesion, the PI3K-Akt signalling pathway, and ECM-receptor interaction. Risk scores were correlated with tumor microenvironment (TME) scores, immune cell infiltration, and sensitivities to both chemotherapy and immunotherapy.

**Conclusion:** We have formulated a risk-assessment model pinpointing 13 central HPV-associated genes in BC. These genes present potential as prognostic indicators and therapeutic targets, emphasizing the intertwined relationship between HPV-induced BC progression and the immune landscape.

## Introduction

Bladder cancer (BC) is one of the most common types of cancer worldwide, with about 550,000 new cases and 200,000 deaths reported annually, accounting for 2.1% of all cancer-related deaths 1. According to the latest GLOBOCAN data, BC is particularly prevalent in industrialized nations. For instance, in the United States, BC is the sixth most commonly diagnosed neoplasm 2. Lifestyle-related or environmental carcinogens contribute to around 50% of BC cases 3. On a positive note, globally BC mortality has seen a decline, even with a rise in incidence. However, certain countries like Iceland, Ecuador and Philippines have witnessed an uptick in mortality rates 4, emphasizing the persistent and complex d challenge of combating BC.

Research indicates that the presence of certain microorganisms in the urinary tract is notably associated with genitourinary cancers 5. Patients with bladder cancer tend to have less diverse bladder microbiota and a higher prevalence of certain bacteria compared to healthy individuals 6. For example, BC patients had higher levels of *Fusobacterium* and *Actinobaculum*, while healthy individuals had higher levels of *Veillonella* and *Streptococcus* in their urine 7,8. Researchers also found that individuals at an increased risk of BC recurrence or progression had greater bacterial richness, indicating a potential correlation between microbial composition, richness, and the onset and prognosis of BC 9. Another study involving 57 BC patients and 49 patients with upper urinary tract stones or bladder outlet obstruction found various viruses, including human cytomegalovirus, Epstein-Barr virus, human herpesvirus-6 and human papillomavirus (HPV) in the urine. Notably, significant disparities in HPV viruria rates were observed between cancer patients and the control group 10.

HPV is a DNA virus that targets basal epithelial cells, either on the skin or in mucosal tissues 11. WHO data suggests that 9% to 13% of the global population is infected with HPV, resulting in over 6.2 million positive cases annually in the United States alone 12. Notably, "High-risk" genotypes such as 16, 18, and 31, are implicated in 4.5% of all new cancer diagnoses worldwide, particularly affecting the cervix, anogenital tract (including vulvar, vaginal, anal, and penile), and head and neck regions (like the oropharynx, oral cavity, and larynx) 12. Considering the vicinity of the genital and urinary tract, there's a heightened risk of HPV leading to urinary tract infections 13. A meta-analysis encompassing 27 datasets highlighted a significant correlation between HPV infection and an elevated risk of BC among Asians (OR 6.289, 95% CI 2.167-18.250) 14. Based on a Mendelian random study that consolidated data from 80 articles, there appears to be a potential causal link between HPV infection and the progression of bladder cancer, resulting in an unfavourable patient prognosis 15. Pathological tissue sequencing from 146 bladder cancer patients, as highlighted by Yan et al., revealed that HPV infection, especially strains 18, 33, and 16, is likely a leading causative factor for bladder cancer 16. Above all, HPV infection seems to play a fundamental role in BC onset and advancement. Emphasis should now be placed on devising new biomarkers related to this mechanism, which could assist in early BC diagnosis, prognosis estimation, and therapeutic evaluation.

In this study, we identified genes linked to both bladder cancer prognosis and HPV infection using bioinformatics methods. Based on these findings, we developed an ***HPV-Related Risk Model*** and confirmed its effectiveness in predicting prognosis. Furthermore, we explored the correlations between the risk score, immune cell infiltration, immune cell functional scores, and chemotherapeutic drug sensitivity to elucidate the mechanisms underlying how HPV contributes to the progression of bladder cancer.

## Materials and Methods

### Data acquisition

Sequencing and clinical data were obtained from 431 BC patients through TCGA (https://tcga-data.nci.nih.gov/tcga), which included both cancerous and adjacent tissues. This collected clinical data was inclusive of a range of variables, encompassing age, sex, stage, grade, TNM staging, survival time, and survival status. Following the exclusion of samples characterized by incomplete clinical data, we refined our dataset to include comprehensive clinical information for 409 patients. For validation purposes, dataset GSE13507 was retrieved from GEO (https://www.ncbi.nlm.nih.gov/geo/), encompassing expression profiles for 165 samples.

### Identification of differentially expressed HPV-related genes in BC

18 HPV-related gene sets were extracted from the GSEA database, comprising a total of 1796 HPV-related genes. A comparison of normal and BC samples was performed using the "limma" package in R software, resulting in 429 differentially expressed HPV-related genes. Ultimately, these findings were visualized in a heatmap for HPV gene expression.

### Construct HPV-related prognostic risk model

To identify prognostic significant HPV-related genes, the HPV-related genes obtained from the GSEA database were authenticated by univariate Cox regression. LASSO regression was employed to determine the optimal subset of genes for inclusion in the risk model. Eventually, the multiple Cox regression and AIC value were applied to establish a prognostic risk model.

### Evaluate the prognostic HPV related risk model

The “survival” and “survminer” packages in the R language were employed to perform the Kaplan-Meier survival analysis across the entire dataset, as well as for the subsets of clinical stage I-II and clinical stage III-IV sets. Additionally, the accuracy and diagnostic efficacy of the prognostic model were evaluated using the receiver operating characteristic (ROC) curve and the area under the curve (AUC).

### Functional and pathway enrichment analyses

DEGs (differentially expressed genes) were obtained by comparing the expression between the high-risk and low-risk groups. Using Gene Ontology (GO) enrichment analysis DEGs were annotated in terms of Annotation of cellular components (CCs), biological processes (BPs), and molecular functions (MFs). To identify the route of gene clusters and their associated functions, the Kyoto Encyclopedia of Genes and Genomes (KEGG) pathway analysis (http://www.genome.jp/) was employed. Additionally, further functional enrichment analysis was performed with the aid of the database for annotation, visualization, and Integrated Discovery (DAVID) (https://DAVID.ncifcrf.gov/).

### TME cell infiltration

To quantify the immune and stromal components within tumors, the “ESTIMATE” package was utilized to determine the Cancer purity scores. To understand the proportion and prognostic significance of immune cells, the genetic expression data was analyzed using the online platform CIBERSORT via LM22 signature leveraged with 1,000 permutations. Subsequently, bar and box plots were generated to visualize the findings.

### Drug sensitivity analysis

To forecast drug susceptibility differences between high-risk and low-risk groups, data from cellminer (https://discover.nci.nih.gov/cellminer/home.do) were utilized and analyzed by the 'rcellminer' R package. Subsequently, the predictive power of the constructed signature for immunotherapy efficacy was evaluated using data from the IMvigor210 cohort. Survival analysis was conducted with the 'survival' and 'survminer' R package. Prognostic factors were identified through Cox regression, and the Kaplan-Meier survival curves were compared using the log-rank test. Finally, the correlation between the risk score and efficacy in solid tumor was examined through ROC analysis.

### Real-time quantitative polymerase chain reaction (RT-qPCR)

This project utilized cell lines from established institutes. SV-HUC-1 (SV1) and T24, both from the Center for Excellence in Molecular Cell Science, Chinese Academy of Sciences; J82 and 5637, representing bladder transitional cell carcinoma, from the Cell Resource Center, Chinese Academy of Medical Sciences; and UM-UC-3 (UC3), a bladder cancer line from Wuhan, China. RT-qPCR was employed to validate the expression levels of select genes from the ***HPV-Related Risk Model*** in both bladder cancer cell lines (T24, J82, UC3, 5637) and normal bladder cell lines (SV1) 17. To assess the differences between these groups, we conducted a Kruskal-Wallis test using GraphPad Prism software (Version 9.5.0). The primer sequences can be found in the Supplementary Table 1.

### Cell line construction and RNA transcriptome sequencing (RNA-seq)

This study utilized transcriptome sequencing to explore and validate HPV-related risk model genes. First, we transfected all mentioned bladder cell lines with lentiviral vectors which were pLV[Exp]-CMV>{HPV16E6:HPV16E7/3xFLAG}-EF1A>{CopGFP:T2A:Puro} to construct HPV16E6E7 high-expression cell (HPV-cell). Subsequently, cisplatin-resistant (CR) bladder cancer cell lines were developed from J82 and T24 through cisplatin concentration screening to 1.5 μg/ml. RNA sequencing (RNA-seq) was then conducted to analyze gene expression differences under various experimental conditions. Total RNA was extracted from the cell samples using TRIzol reagent, followed by quality control with the Agilent 2100 system. Libraries were constructed, and high-throughput sequencing was performed on the Illumina platform, generating substantial raw sequence data. FastQC and Trimmomatic were employed for quality control and filtering of the raw data. The cleaned sequences were aligned to the reference genome, and gene expression levels were analyzed using R packages, including TCGAbiolinks, pheatmap, and DESeq2, to identify differentially expressed genes. DNA/RNA/Small RNA/cDNA library sequencing was performed on the Illumina Novaseq X Plus by Gene Denovo Biotechnology Co, Ltd (Guangzhou, China).

### Statistical analysis

In this study, all data analyses were executed using R language (Version 4.2.2). Statistical significance was defined as a p-value below 0.05 unless otherwise stated.

## Results

### Differential expression analysis of HPV-related genes

**Figure 1:**
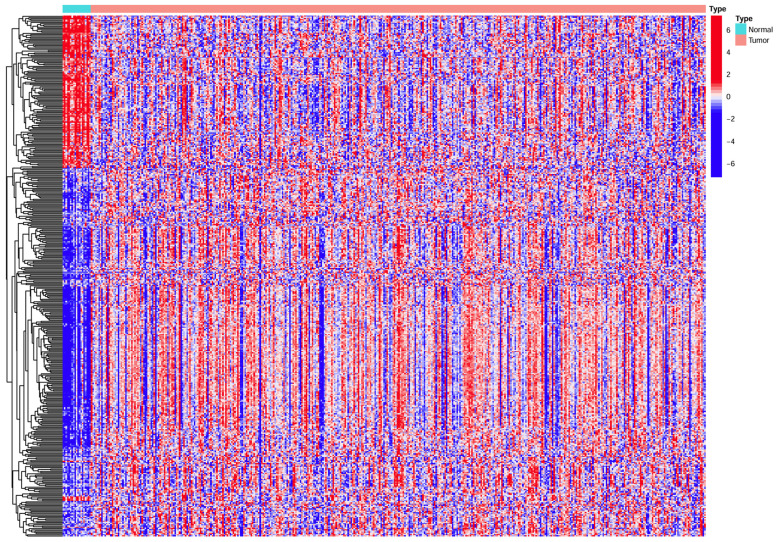
** Heatmap showing HPV-related gene expression in BC samples versus normal samples.** Horizontal coordinates represent samples, while vertical coordinates correspond to HPV-related genes represent.

The expression of HPV-related genes was analyzed in 409 BC samples and 22 normal bladder samples. The heatmap (Figure 1) from TCGA revealed the differential expression of HPV-related genes between BC and normal samples.

### Construct prognostic HPV-related risk model

**Figure 2:**
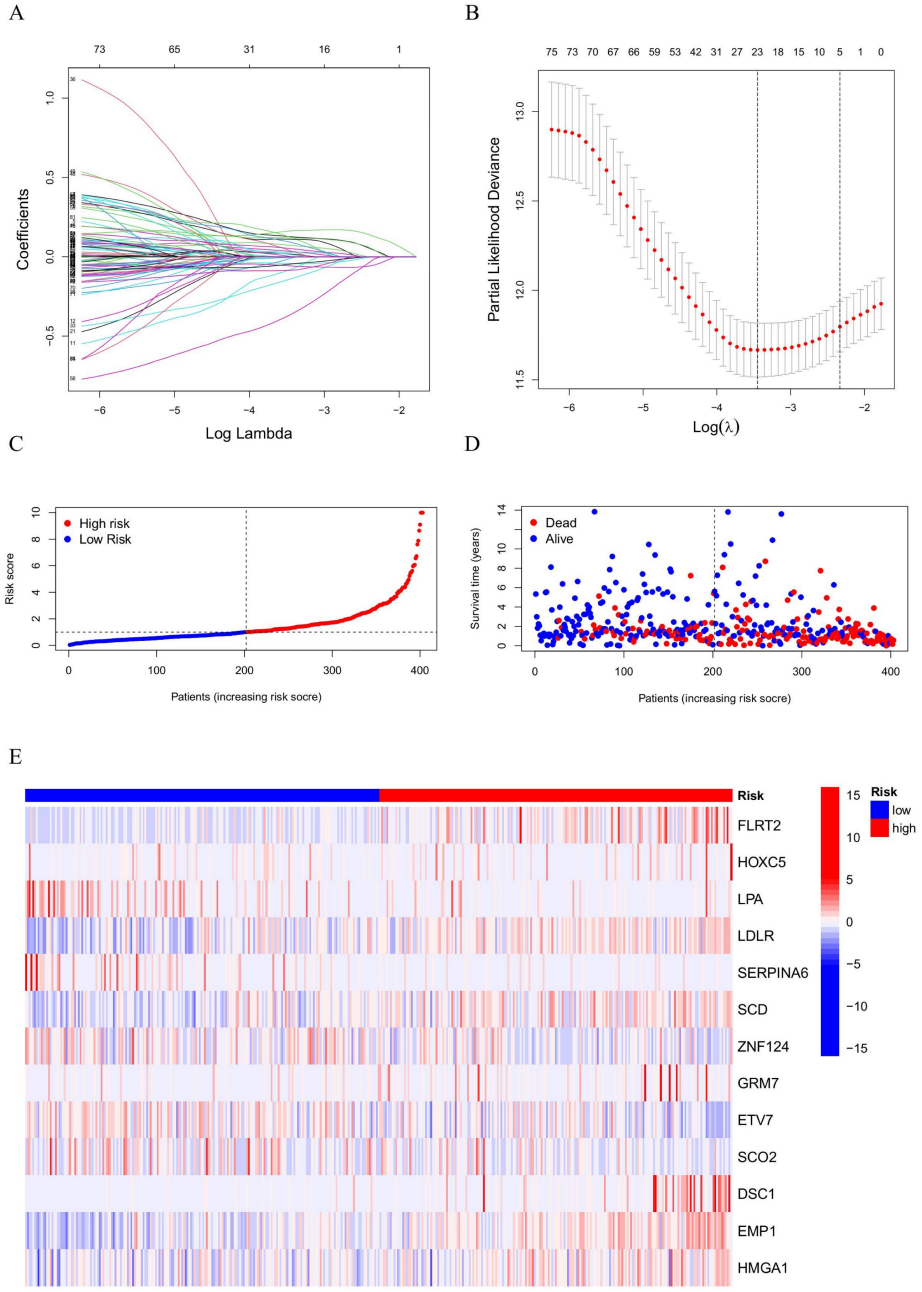
**(A, B)** LASSO result diagram of TCGA set and **(C, D)** Risk curves and survival scatter plots of BC patients and **(E)** Heatmap of 13 HPV-related genes expression.

Using data from BC samples, univariate Cox regression was applied and identified 50 prognostic HPV-related genes. To refine the model, lasso regression was utilized, narrowing the list to 23 genes suitable for further model construction (Figure 2A, B). Using multivariate Cox regression analysis, 13 genes were identified for the HPV-related risk model. The outcomes revealed that FLRT2, HOXC5, LPA, LDLR, SERPINA6, SCD, ZNF124, GRM7, ETV7, SCO2, DSC1, EMP1, HMGA1 had an independent effect on prognosis of BC patients. The formula followed was used to calculate the risk score:

Risk Score = (EXP FLRT2×0.232408459255271)+(EXP HOXC5×0.269302765402229)+(EXP LPA×-0.395660601925108)+(EXP LDLR×0.111276756609973)+(EXP SERPINA6×-0.361065990663277)+(EXP SCD×0.136025004656667)+(EXP ZNF124×-0.279724171939346)+(EXP GRM7×0.323739304828735)+(EXP ETV7×-0.101661530874702)+(EXP SCO2×-0.602292793435388)+(EXP DSC1×0.155792329376409)+(EXP EMP1×0.212386153563448)+(EXP HMGA1×0.216909472643139)

EXP represents the level of HPV-related gene expression. BC patients were categorized into high-risk and low-risk groups based on their median risk score (Figure 2C). The survival state scatter plot revealed a compromised survival status for patients with high-risk scores (Figure 2D). Genes involved in the risk model were significantly expressed differently between high-risk and low-risk groups (Figure 2E). Specifically, FLRT2, HOXC5, LDLR, SCD, GRM7, DSC1, EMP1, HMGA1 were identified as risk factors, while LPA, SERPINA6, ZNF124, ETV7, SCO2 were deemed protective.

### Evaluation of prognostic HPV-related risk model

**Figure 3:**
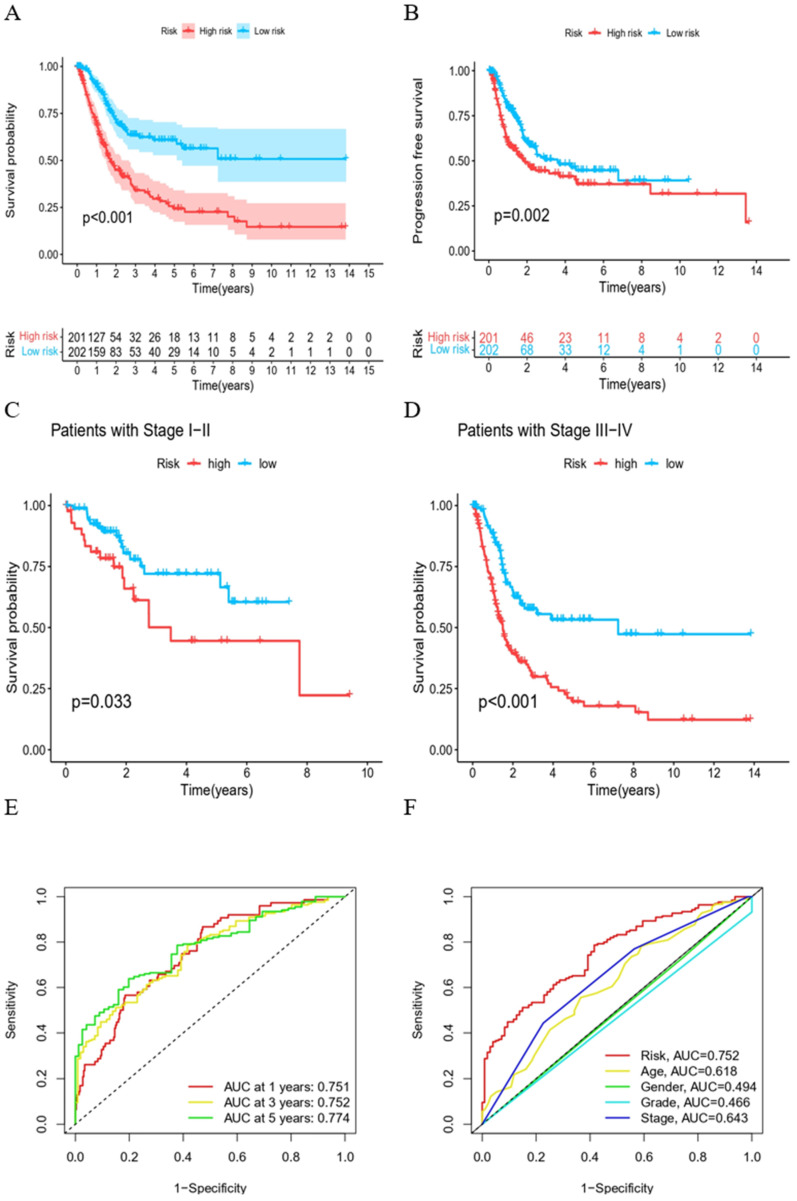
** Evaluation of prognostic risk models for HPV-related genes. (A)** Kaplan-Meier survival curves of two groups of BC patients. **(B)** Survival curves of PFS of two groups of BC patients. **(C)** Survival curves of BC patients during stage I-II. **(D)** Survival curves of BC patients during stage III-IV. **(E)** ROC curves of predictive efficacy to 1, 3, 5 years. **(F)** ROC curves of HPV-related risk model and other clinical prognostic factors.

Kaplan-Meier survival curves demonstrated that both overall survival (OS) and progression-free survival (PFS) were significantly shorter in the high-risk group (Figure 3A, B). Irrespective of being in the high or low clinical stage groups, a diminished overall survival was evident in the high-risk group (Figure 3C, D). Subsequently, ROC curves were employed, evaluating 1-, 3-, and 5-year OS for BC patients. AUC values of 0.751, 0.752, and 0.774 were recorded, respectively, suggesting the HPV-related gene expression risk model is reliable in predicting BC patient prognosis (Figure 3E). Furthermore, the superiority of this risk score over other established clinical prognostic indicators was emphasized by the ROC curves (Figure 3F).

### Verification of the predictive efficacy of HPV-related risk model

**Figure 4:**
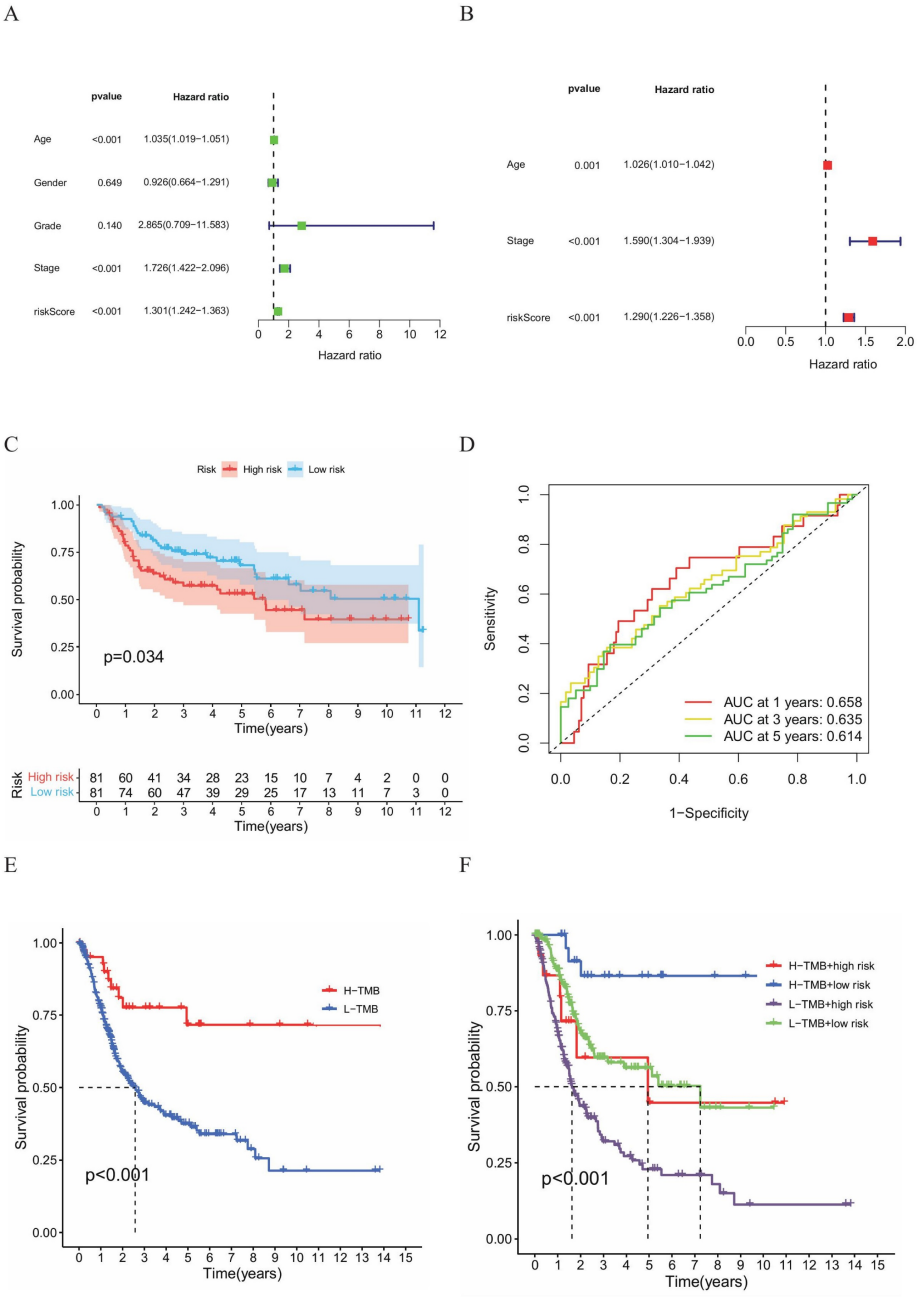
**(A)** Univariate and **(B)** multivariate Cox regression analyses of the relationship between multiple clinical variables (including risk scores). **(C)** Kaplan-Meier survival curves for both groups of BC patients. **(D)** ROC curves to assess the predictive efficacy of the risk model. **(E)** Kaplan-Meier survival analysis of the H-TMB group and L-TMB group and **(F)** Kaplan-Meier survival analysis of the H-TMB high-risk group, H-TMB low-risk group, L-TMB high‑risk group and L-TMB low-risk group.

To determine whether the HPV-Related Risk Model independently predicts OS in BC patients, both univariate and multivariate Cox regression were performed (HR:1.301, 95%CI: 1.242-1.363; HR: 1.290, 95%CI: 1.226-1.358, Figure 4A, B). The univariate Cox regression analysis identified age, stage and risk as risk factors. The multivariate Cox regression highlighted that risk score, age and stage as independent predictors. The predictive performance of the HPV-related risk model was substantiated in BC cohort analysis from GEO database. Kaplan-Meier survival curves revealed a shorter OS for high-risk individuals (Figure 4C). Additionally, ROC curves evaluated the risk model's 1-, 3-, and 5-year accuracy, yielding AUC values of 0.658, 0.635, and 0.614 respectively (Figure 4D). These outcomes aligned with findings from the TCGA database, suggesting the risk score had certain accuracy as a prognostic predictor of BC patients.

### Analysis of the combined gene and clinical model

A noteworthy disparity e in tumor mutation burden (TMB) was observed between the two HPV-related risk groups, with the high TMB group exhibiting a better prognosis than its low TMB counterpart (Figure 4E). Extending these findings, we additionally ascertained that high-risk groups, categorized using the same calculation formula as the training set, were linked to poorer prognosis in the validation sets (Figure 4F). To further refine our prognostic assessment, we integrated risk scores with TMB levels. Our analysis revealed that, irrespective of TMB levels, the high-risk group faced lower survival rates compared to the low-risk group. Noteworthy, the high-risk group with low TMB exhibited a survival probability comparable to that of the low-risk group with high TMB.

### Exploration of HPV-related risk model genes expression using RNA-seq

**Figure 5:**
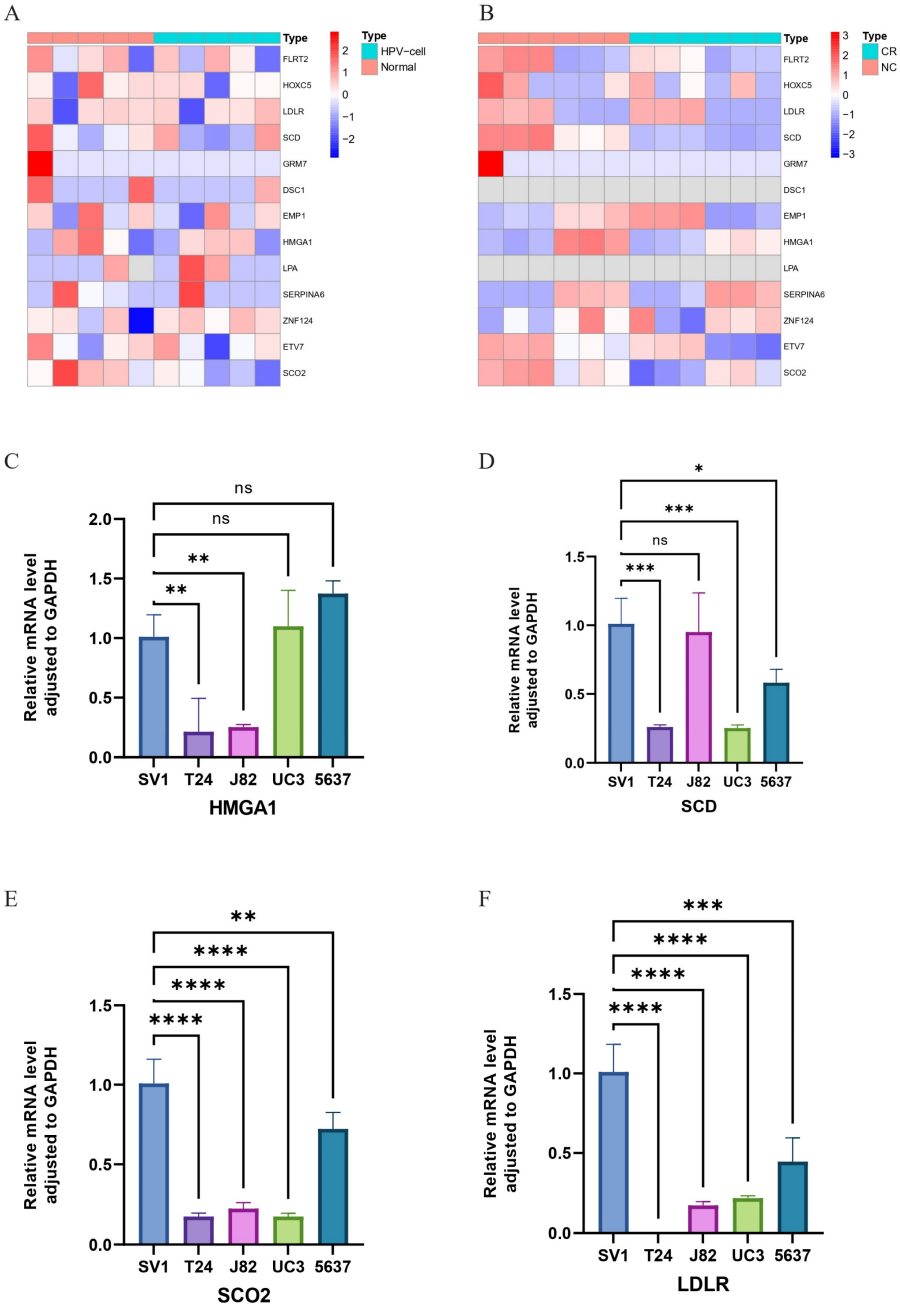
** Heatmap of gene expression based on RNA-seq. (A)** Between normal bladder cells and corresponding HPV-cells. **(B)** Between the wild-type bladder cancer (NC) strain and the bladder cancer cisplatin-resistant (CR) strain **(C-F)** Relative mRNA level adjusted to GAPDH of HMGA1, SCD, SCO2 and LDLR in SV-HUC-1, T24, J82, UM-UC3 and 5637 cell lines through RT-qPCR. ns ? **** ?? ***p < =0.001 **p <= 0.01 and *p <=0.05.

We utilized RNA-seq to analyze the expression levels of 13 HPV-related risk model genes in HPV-transfected cell lines compared to normal cell lines, as well as in bladder cancer cell lines compared to cisplatin-resistant bladder cancer cell lines. The results revealed that in HPV-transfected cells, FLRT2, HOXC5, LDLR, SCD, GRM7, DSC1, ETV7, and SCO2 were significantly upregulated, whereas EMP1, HMGA1, LPA, SERPINA6, and ZNF124 were notably downregulated compared to their expression in corresponding normal cells (Figure 5A). In cisplatin-resistant bladder cancer cell lines, FLRT2, HOXC5, SCD, GRM7, HMGA1, ETV7, and SCO2 were highly expressed, while EMP1, SERPINA6, and ZNF124 exhibited lower expression levels (Figure 5B). These findings suggest that HPV-related risk model genes may play a critical role in the progression and therapeutic response of bladder cancer.

### Validation of HPV-related risk model genes expression using RT-qPCR

**Figure 6:**
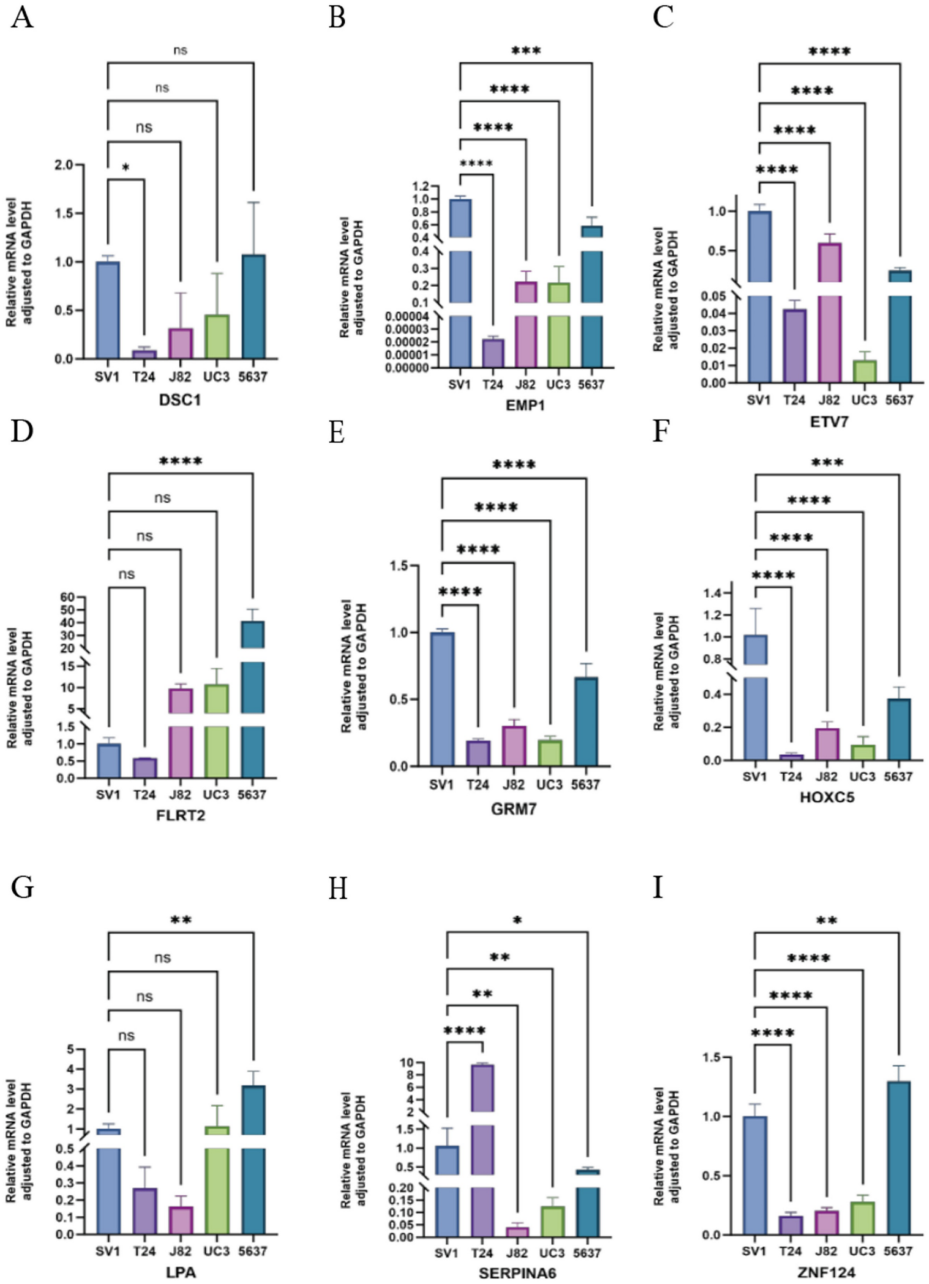
Relative mRNA level adjusted to GAPDH of DSC1, EMP1, ETV7, FLRT2, GRM7, HOXC5, LPA, SERPINA6 and ZNF124 in SV-HUC-1, T24, J82, UM-UC3 and 5637 cell lines through RT-qPCR. ***p < =0.001 **p <= 0.01 and *p <=0.05.

We utilized RT-qPCR to validate the expression levels of HMGA1, SCD, SCO2, LDLR, DSC1, EMP1, ETV7, FLRT2, GRM7, HOXC5, LPA, SERPINA6, and ZNF124 in the SV-HUC-1 cell line, bladder tumor cell lines (T24, J82, UC3, 5637), and normal bladder tissues. Notably, FLRT2 and LPA were significantly upregulated in bladder tumor cell lines compared to normal bladder cells, suggesting a role in tumor progression. In contrast, SCD, SCO2, LDLR, EMP1, ETV7, GRM7, HOXC5, and ZNF124 were significantly downregulated, indicating potential tumor-suppressor functions. The expression of HMGA1 and SERPINA6 remained relatively stable, suggesting their roles might depend on environmental factors, while minimal changes in DSC1 could indicate subtype-specific effects (Figures 5C-F and 6). These findings support the bio-informatics analysis and highlight the potential roles of these genes in bladder cancer progression.

### Functional and pathway enrichment analyses

**Figure 7:**
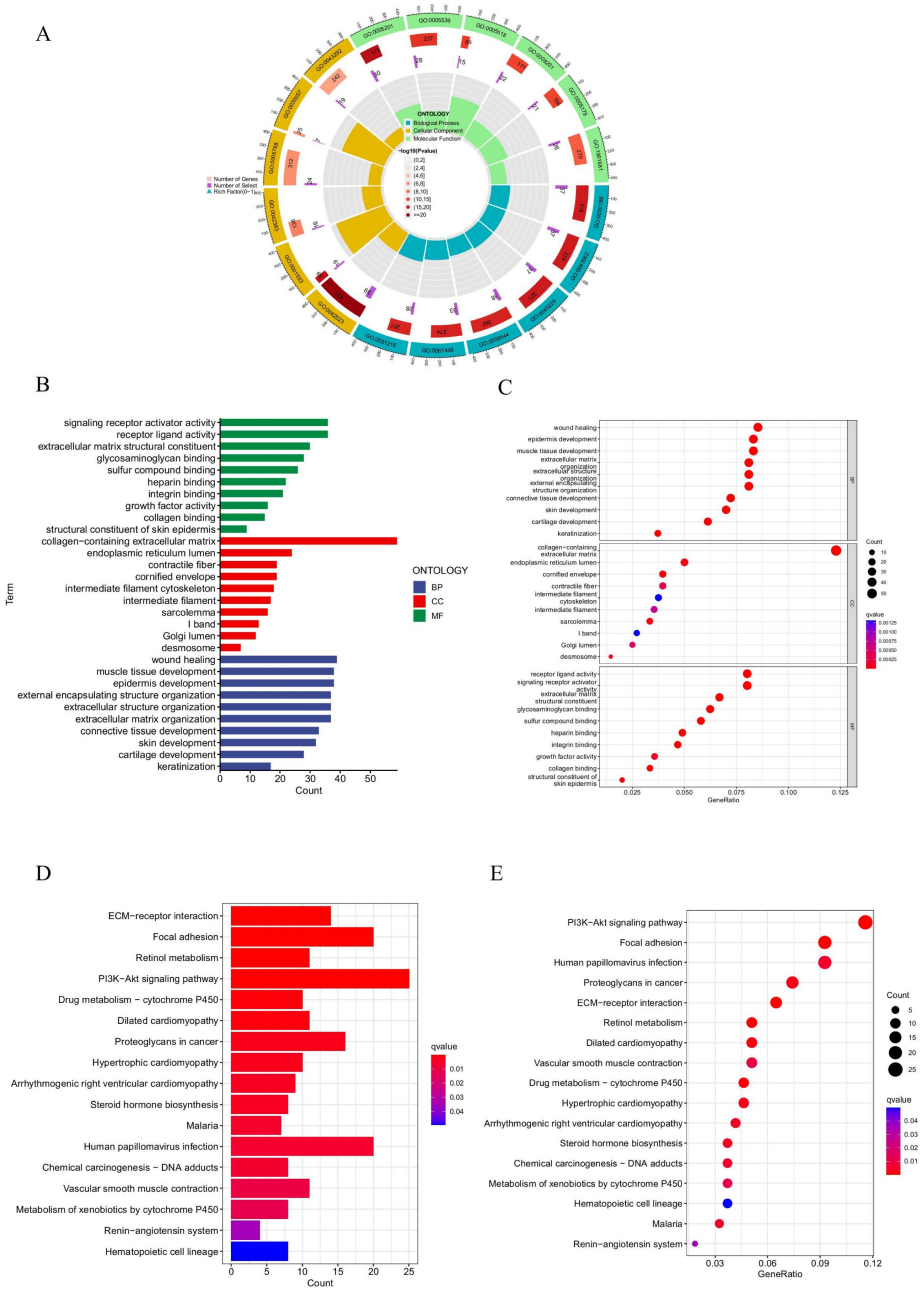
** GO and KEGG analysis. (A, B, C)** GO analysis on the biological processes (BP), cellular components (CC), and molecular functions (MF). **(D, E)** The KEGG pathway enrichment analysis.

To explore the underlying biological mechanisms of HPV in BC progression, GO functional and KEGG pathway enrichment analyses were performed. The GO analysis results revealed that these prognostic HPV-related genes were predominantly enriched in biological processes (BPs) related to wound healing, epidermis development, external encapsulating structure organization, extracellular structure organization, and extracellular matrix organization. In terms of molecular function (MF), these genes were primarily associated with extracellular matrix structural constituents, collagen binding, glycosaminoglycan binding, and sulfur compound binding. Regarding cellular components (CCs), the genes were mainly enriched in the collagen-containing extracellular matrix, endoplasmic reticulum lumen, cornified envelope, and desmosome (Figures 7A-C). The KEGG pathway analysis revealed primary enrichment in the focal adhesion, PI3K-Akt signaling pathway and ECM-receptor interaction (Figures 7D, E).

### Correlation between prognostic HPV-related risk model and immune cell infiltration

**Figure 8:**
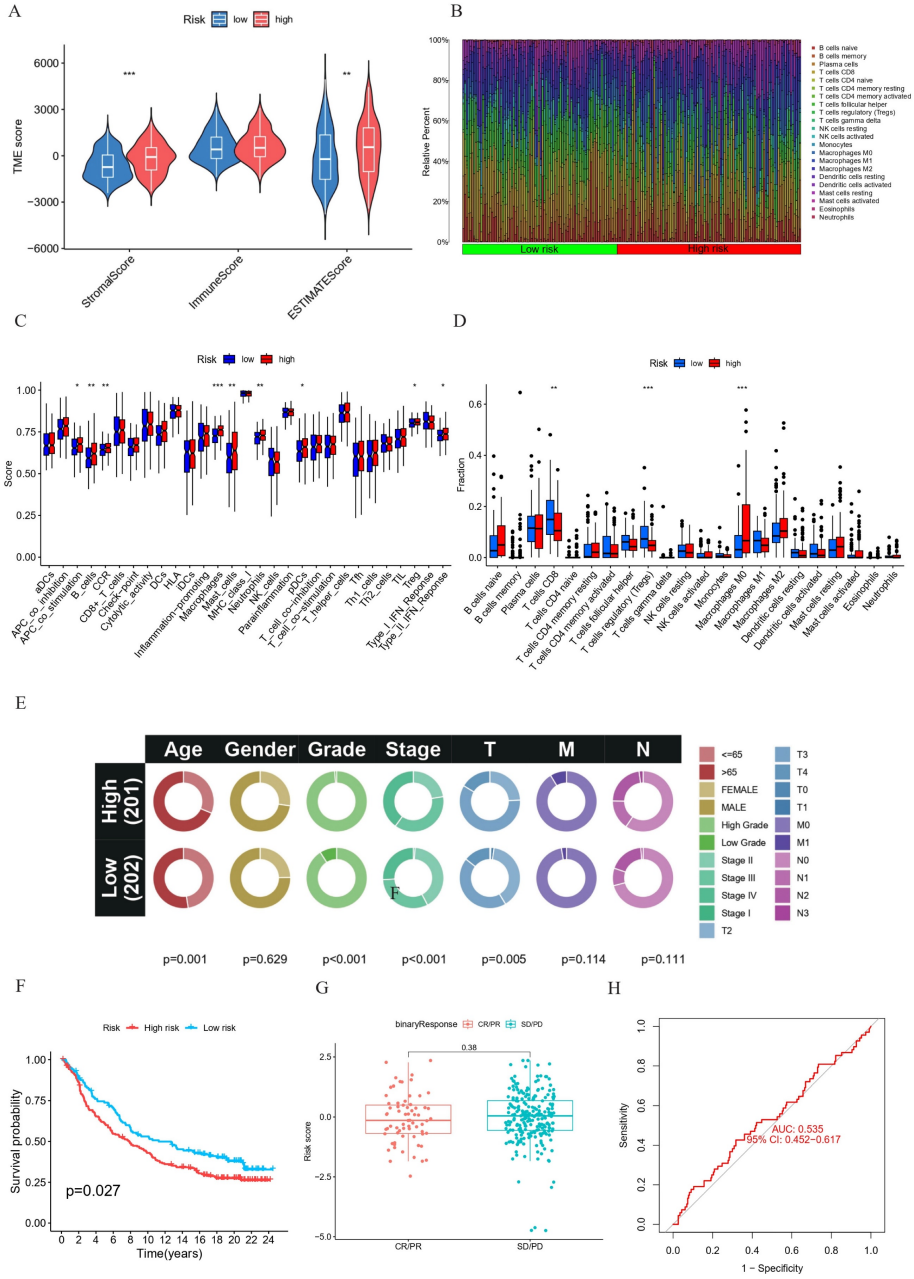
**(A-B)** TME immunocyte infiltration characteristics and immune constituents in different risk groups. **(A)** Comparative analysis of stromal, immunity, and estimate scores between high-risk and low-risk groups. **(B)** Bar plot representing the differential proportions of 22 types of immune cell between high-risk and low-risk groups. **(C-D)** Immunocyte infiltration in different risk groups. (C) Boxplot for the immune-associated functions. (D) Boxplot of the immune cell abundance. (*p < 0.05; ** p < 0.01; *** p < 0.001) E-H Univariate and multivariate Cox regression analysis of the correlation between OS and various clinical variables **(E)**. Kaplan-Meier survival analysis **(F)** and correlation between the risk score and clinical response (complete response [CR]/partial response [PR] and stable disease [SD]/progressive disease [PD]) **(G)** and ROC curve **(H)** in the IMvigor210 cohort. ***p < =0.001 **p <= 0.01 and *p <=0.05.

The immune microenvironment, comprising fibroblasts, extracellular matrix, immune cells, various growth factors, inflammatory factors and cancer cells, plays a critical role in tumor progression. The results revealed statistically significant higher stromal and ESTIMATE scores in the high-risk group (Figure 8A). To further employ the CIBERSORT method, a support vector regression-based deconvolution algorithm. Compared the type and proportion of 22 types of immune cells between the two groups (Figure 8B), we found that high-risk BC patients exhibited increased infiltration of Macrophages M0 and decreased infiltration of T cells regulatory (Tregs) and T cells CD8 (Figures 8D). These findings establish a correlation between risk scores and immune cell infiltration, suggesting an association between HPV-related risk groups among BC patients and immune cell infiltration. Additionally, a significant increase in immune function scores was observed in high-risk patients with B cells, macrophages, mast cells, neutrophils, DCs and Treg (Figure 8C). Collectively, these results indicate the influence of prognostic HPV-related genes on BC progression potentially is via modulation of the tumor immune microenvironment.

### Verification of the HPV-related risk model in the IMvigor210 cohort

In the IMvigor210 cohort, we conducted a correlation analysis to examine the relationship between HPV-related risk scores and various clinical characteristics of patients, such as age, gender, grade, and stage. These findings are visually represented in circos graphs (Figure 8E). Subsequent Kaplan-Meier analysis revealed that patients in the high-risk score category face a less favorable prognosis, as illustrated in (Figure 6 F). Despite these insights, our model's limitations become apparent when considering chemotherapy responsiveness. The box-and-whisker plots and ROC curves for chemotherapy responsiveness closely mirror those of the risk score model, suggesting that our model may not be well-suited for predicting immunotherapy outcomes in this specific cohort (Figure 8G, H).

### Drug sensitivity analysis

We analyzed the relationship between the drug response and the expression of key biomarkers, FLRT2, LDLR, EMP1, ZNF124, HMGA1, LPA and SERPINA6 through the CellMiner database. Our findings reveal that the expression of 12 biomarkers was significantly and negatively correlated with drug sensitivity (Supplement Figure 1). Specifically, FLRT2, LDLR, EMP1, and SERPINA6 exhibited a negative correlation with drug sensitivity, while ZNF124, HMGA1, and LPA showed a positive correlation. These findings serve as important reference points for individualizing treatment plans for patients with BC.

## Discussion

Currently, BC is a major global health concern with a high incidence and poor prognosis 18. Despite the application of new therapies in clinical settings, BC remains a challenging disease, necessitating the discovery of new biomarkers for screening, diagnosis, and prognosis 18,19. Previous studies have found a correlation between HPV and cervical cancer, anal-genital cancer, and head and neck cancer 20. However, the association between HPV and bladder cancer remains a topic of debate due to regional disparities and variations in detection techniques 21. Recent publications are increasingly suggesting that HPV may not only be involved in initiating bladder cancer but also play a fundamental role in BC progression 15. In this study, we applied bioinformatics methods to identify 13 core HPV-related genes in BC genes from TCGA and GEO. We then developed and pathologically validated a prognostic risk score that predicts both patient prognosis and drug sensitivity. To our knowledge, this study is the first of its kind to address these issues.

This study identified 13 core genes associated with HPV-related BC. Among these, FLRT2, HOXC5, LDLR, SCD, GRM7, DSC1, EMP1, and HMGA1 are classified as risk genes. In contrast, LPA, SERPINA6, ZNF124, ETV7, and SCO2 are considered protective effect genes. The fibronectin leucine-rich transmembrane protein 2 (FLRT2) is known to mediate endothelial adhesion in tumors, potentially enhancing the invasive characteristics of colorectal cancer cells 22. However, another study suggests FLRT2 might induce ferroptosis, which can inhibit BC progression 23. The overexpression of Homeobox (HOX) family genes has been linked to poor prognosis in multiple cancers, with HOXC5 identified as a risk factor for pediatric gliomas and renal clear cell carcinoma 24, 25. The low-density lipoprotein receptor (LDLR) plays a pivotal role in the RAS/RAF/MAPK(MEK)/ERK pathway triggered by low-density lipoproteins. Its association with thyroid cancer progression is well-documented 26. Stearoyl-CoA desaturase 1 (SCD1) overexpression has been detected in various cancers, with elevated levels leading to increased invasiveness in malignancies such as oesophagal squamous cell carcinoma 27, 28.

The glutamate receptor 7 (GRM7) is proposed to predict prognosis in head and neck cancers as well as glioblastomas 36010616, 35626148. Moreover, a study focusing on African American prostate cancer patients found a significant association between differential GRM7 methylation and disease progression 29. Multiple studies have linked Desmocollin-1 (DSC1) with enhanced migration and invasion abilities in breast cancer cells 30, 31. Research has shown that the epithelial membrane protein 1 (EMP1) plays a critical role in metastatic relapse of colorectal cancer, and Zhou et al. found its impact on the prognosis and immunotherapy outcomes in BC patients 32, 33. Lastly, High mobility group A1 (HMGA1) demonstrates elevated expression in various malignant tumors, including pancreatic ductal adenocarcinoma and hepatocellular carcinoma, suggesting its role in enhancing cancer cell invasiveness 34-36. In summary, while many of the 8 risk genes we identified are known risk factors in other cancers, their roles in BC require further exploration. Lysophosphatidic acid (LPA) receptor-mediated signaling is pivotal in the regulation of cell proliferation and differentiation. Studies in non-small cell lung cancer animal models have confirmed that elevated LPA expression can diminish anti-tumor immunity, thereby reducing the efficacy of immunotherapy 37, 38. A sequencing study on myeloma revealed that the Zinc Finger Protein 124 (ZNF124) is predominantly expressed in the most aggressive tumor subgroups, correlating with an adverse prognosis 39. In breast cancer, the ETS (E26 transformation-specific) transcription factor 7 (EVT7) has been identified to temper the inflammatory response by inhibiting the TNFR 1/NF-κB axis, a crucial determinant of drug resistance 40, 41. Supporting this, Kan et al. observed a negative association between the prognosis of muscle-invasive BC and the heightened expression of ETV7-AS1 42, aligning with our findings. The Synthesis of Cytochrome C Oxidase 2 (SCO2) is postulated to be a downstream effector of Tumor Protein P53 (TP53), modulating cellular respiration and metabolism to counteract tumor progression 43. Presently, while research on SERPINA6 predominantly centers around endocrine disorders and cardiovascular risks, its role in oncology is yet uncharted 44, 45. In conclusion, among the 13 core HPV-related BC genes we identified, the majority have been investigated in other malignancies, but their specific roles in BC await exploration, offering promising molecular targets for subsequent studies.

Based on the GO functional enrichment analysis, we postulate that BC cells may modulate tumor invasion and metastasis by governing the formation of extracellular matrices and inducing apoptosis in epithelial cells. Our KEGG analysis primarily identifies three key functional pathways: focal adhesion, the PI3K-Akt signaling pathway, and ECM-receptor interaction. Focal adhesions, specialized structures at the cell-extracellular matrix junctions, are critical in upholding cell tone and mediating signal transduction that fosters cell survival. Research indicates that molecules related to focal adhesion influence the epithelial-mesenchymal transition (EMT), as well as the invasion and metastasis of tumor cells 46-48. Furthermore, the PI3K-Akt signaling pathway, integral in various cancers, orchestrates a plethora of processes including cancer cell proliferation, apoptosis, and metastasis 49. In the tumor milieu, this pathway also contributes to angiogenesis and the mobilization of inflammatory agents 50, 51. Crucially, evidence suggests that the PI3K/Akt pathway plays a pivotal role in the mediation of the EMT process, presenting itself as a potential therapeutic target for metastatic tumors 52. The ECM, a rich three-dimensional macromolecular lattice, is a predominant constituent of the tumor microenvironment 53. Acting as a nexus, the ECM directs cell differentiation, migration, and invasion, consequently shaping the behaviour of tumor cells 54. Supporting this notion, Brooks et al. posited that alterations in type I collagen within the ECM milieu might be pivotal in the evolution of non-muscular invasive bladder cancer 55. Conclusively, we hypothesize that prognostic HPV-related genes in BC cells may amplify the survival, migratory, and invasive capabilities of tumor cells via the EMT mechanism. Targeting the EMT process and curtailing ECM-receptor interactions might offer promising avenues to enhance bladder cancer prognosis.

In this study, we examined the association in immune cell infiltration across high-risk and low-risk individuals, revealing a significant difference in the distribution patterns of tumor-infiltrating immune cells (TIICs). According to the immunoscore findings, the ratio of immune components to matrix components within the tumor microenvironment (TME) correlates with prognosis patterns in high-risk and low-risk patients. Notably, differences in matrix cells were more pronounced than in immune cells. This observation suggests that the immunological variations between high-risk and low-risk groups are primarily evident in the matrix cells. Higher assessment scores are associated with an increased risk in the prognosis model and, consequently, worse patient outcomes. An analysis of immune infiltration revealed that the high-risk group exhibited higher levels of M0 macrophages but lower levels of Tregs and CD8^+^ T cells, both of which are recognized as tumor-suppressive cells in malignant tumors. M0 macrophages, a subtype that can transform into polarized macrophages, can potentially be further cultured into M1 or M2 cells 56. For patients diagnosed with muscle-invasive bladder cancer (MIBC), a higher concentration of M1 macrophages is associated with a better response to immunotherapy. In contrast, an increased presence of M2 macrophages may contribute to immunotherapy resistance 57. Therefore, steering the polarization of tumor-associated macrophages toward M1 may present a valuable therapeutic approach for BC treatment. Furthermore, the study explored the influence of HPV prognosis-related genes on immune cell concentration scores and immune functions. High-risk groups scored higher for B cells, macrophages, mast cells, Ccr, and APC co-stimulation. This elevation in scores could be a contributing factor to the varying effects of immunotherapy observed among patients with different risk profiles.

A higher Tumor Mutational Burden (TMB) enhances the immunogenicity of tumor cells, making them more easily recognized by T cells. This improved recognition facilitates a more effective immune-killing mechanism, potentially leading to greater benefits from immunotherapy. Our results indicate that among both high-risk and low-risk groups of BC patients, those with higher TMB tend to have a better prognosis. This favorable prognosis may be attributed to the enhanced efficacy of immunotherapy in patients with a high TMB.

In this study, correlations were identified between the sensitivity of drugs and the expression levels of specific biomarkers. Initially, we noted that Belinostat, an effective and relatively tolerable agent, has demonstrated efficacy in treating superficial urinary bladder cancer by inducing both growth inhibition and cell cycle arrest 58. Additionally, the combination of Palbociclib, a known CDK4/6 inhibitor, with Talazoparib is acknowledged as a vital treatment for BC 59. Our analysis revealed that an upregulation of FLRT2 and EMP1 correlates with diminished sensitivity to Belinostat and Palbociclib. This finding implies that FLRT2 and EMP1 might serve as valuable predictive markers for therapeutic efficacy in chemotherapy treatments. Furthermore, we observed that LDLR upregulation is associated with decreased sensitivity to Oxaliplatin. Oxaliplatin is a medication renowned for inhibiting tumor growth, particularly when combined with anti-PD-1 inhibitors 60. This relationship suggests LDLR may have a similar effect to FLRT2. It is crucial to note that drugs like Nelarabine, Fludarabine, Umbralisib, and Cladribine, typically used against hematopoietic system malignancies, might also possess potential therapeutic effects in BC chemotherapy. According to our findings, a reduction in ZNF124 expression is associated with lower sensitivity to Nelarabine, Palbociclib, and Ifosfamide, while downregulation of HMGA1 correlates with decreased sensitivity to Fludarabine and Cladribine. Consequently, ZNF124 and HMGA1 could be explored as potential targets for BC treatment.

Regarding LPA, it is associated with Imiquimod, which has been proven effective in reducing bladder carcinogenesis by controlling tumor proliferation and apoptosis 61. LPA's connection to Imiquimod, as well as to Megestrol Acetate—a drug commonly used against breast cancer—may be analogous to the relationships observed with ZNF124 and HMGA1. Furthermore, another medication, Eribulin Mesylate, approved for treating metastatic breast cancer and currently under Phase I/II trials for bladder cancer, exhibits a negative correlation with SERPINA6 62, 63. These findings suggest that SERPINA6 may serve as a potential target for Eribulin Mesylate-based therapies.

Despite its contributions, our study does possess certain limitations. Firstly, our findings require further validation through more functional experiments, including studies using animal models and human tissue samples, to facilitate their translation into clinical applications. Secondly, since this study is retrospective and relies on public databases, the clinical effectiveness and stability of our findings must be confirmed with more comprehensive clinical data. Lastly, while our study primarily focuses on genomic-level exploration, additional research integrating proteomics and metabolomics is essential for a deeper understanding of the biological mechanisms underlying the interaction between HPV and BC.

## Conclusion

We developed a risk-scoring model identifying 13 core HPV-related genes in bladder cancer. These genes hold potential as prognostic markers and therapeutic targets for chemotherapy and immunotherapy. Our findings underscore the strong interplay between HPV-driven bladder cancer progression and the immune microenvironment, offering valuable insights into the disease's underlying mechanisms and potential avenues for clinical intervention.

## Supplementary Material

Supplementary figures and tables.

## Abbreviations

BC: Bladder cancer
HPV: Human papillomavirus
CR: Cisplatin Resistant
ROC: Receiver operating characteristic
AUC: Curve and the area under the curve
DEGs: Differentially expressed genes
KEGG: Kyoto encyclopedia of genes and genomes
DAVID: Annotation, visualization, and Integrated Discovery
RT-qPCR: Real-time quantitative polymerase chain reaction
HPA: Human protein atlas
OS: Overall survival
PFS: Progression-free survival
BPs: Biological processes
MF: Molecular function
CC: Cell composition
Tregs: T cells regulatory
TMB: Tumor mutation burden
EMT: Epithelial-mesenchymal transition
TME: Tumor microenvironment
MIBC: Muscle-invasive bladder cancer

## References

[1] Richters A, Aben KKH, Kiemeney LALM (2020). The global burden of urinary bladder cancer: an update. World J Urol.

[2] Lobo N, Afferi L, Moschini M, Mostafid H, Porten S, Psutka SP (2022). Epidemiology, Screening, and Prevention of Bladder Cancer. Eur Urol Oncol.

[3] Laaksonen MA, MacInnis RJ, Canfell K, Giles GG, Hull P, Shaw JE (2020). The future burden of kidney and bladder cancers preventable by behavior modification in Australia: A pooled cohort study. Int J Cancer.

[4] Wong MCS, Fung FDH, Leung C, Cheung WWL, Goggins WB, Ng CF (2018). The global epidemiology of bladder cancer: a joinpoint regression analysis of its incidence and mortality trends and projection. Sci Rep.

[5] Markowski MC, Boorjian SA, Burton JP, Hahn NM, Ingersoll MA, Maleki Vareki S (2019). The Microbiome and Genitourinary Cancer: A Collaborative Review. Eur Urol.

[6] Hrbáček J, Tláskal V, Čermák P, Hanáček V, Zachoval R (2023). Bladder cancer is associated with decreased urinary microbiota diversity and alterations in microbial community composition. Urol Oncol: Semin Orig Investig.

[7] Bučević Popović V, Šitum M, Chow CET, Chan LS, Roje B, Terzić J (2018). The urinary microbiome associated with bladder cancer. Sci Rep.

[8] Yacouba A, Tidjani Alou M, Lagier JC, Dubourg G, Raoult D (2022). Urinary microbiota and bladder cancer: A systematic review and a focus on uropathogens. Semin Cancer Biol.

[9] Wu P, Zhang G, Zhao J, Chen J, Chen Y, Huang W (2018). Profiling the Urinary Microbiota in Male Patients With Bladder Cancer in China. Front Cell Infect Microbiol.

[10] Hrbáček J, Hanáček V, Kadlečková D, Cirbusová A, Čermák P, Tachezy R (2023). Urinary shedding of common DNA viruses and their possible association with bladder cancer: a qPCR-based study. Neoplasma.

[11] Oyouni AAA (2023). Human papillomavirus in cancer: Infection, disease transmission, and progress in vaccines. J Infect Public Health.

[12] Santella B, Schettino MT, Franci G, De Franciscis P, Colacurci N, Schiattarella A (2022). Microbiota and HPV: The role of viral infection on vaginal microbiota. J Med Virol.

[13] Piyathilake CJ, Badiga S, Chambers MM, Brill IK, Matthews R, Partridge EE (2016). Accuracy of urinary human papillomavirus testing for the presence of cervical human papillomaviruses and higher grades of cervical intraepithelial neoplasia. Cancer.

[14] Khatami A, Salavatiha Z, Razizadeh MH (2022). Bladder cancer and human papillomavirus association: a systematic review and meta-analysis. Infect Agents Cancer.

[15] Sun JX, Xu JZ, Liu CQ, An Y, Xu MY, Zhong XY (2023). The association between human papillomavirus and bladder cancer: Evidence from meta-analysis and two-sample mendelian randomization. Journal of medical virology.

[16] Yan Y, Zhang H, Jiang C, Ma X, Zhou X, Tian X (2021). Human Papillomavirus Prevalence and Integration Status in Tissue Samples of Bladder Cancer in the Chinese Population. J Infect Dis.

[17] Huang C, Yang Y, Wang X, Chen S, Liu Z, Li Z (2024). PTBP1-mediated biogenesis of circATIC promotes progression and cisplatin resistance of bladder cancer. Int J Biol Sci.

[18] Sung H, Ferlay J, Siegel RL, Laversanne M, Soerjomataram I, Jemal A (2021). Global Cancer Statistics 2020: GLOBOCAN Estimates of Incidence and Mortality Worldwide for 36 Cancers in 185 Countries. CA Cancer J Clin.

[19] Feng ZH, Liang YP, Cen JJ, Yao HH, Lin HS, Li JY (2022). m6A-immune-related lncRNA prognostic signature for predicting immune landscape and prognosis of bladder cancer. J Transl Med.

[20] De Martel C, Plummer M, Vignat J, Franceschi S (2017). Worldwide burden of cancer attributable to HPV by site, country and HPV type. Int J Cancer.

[21] Yao X, Xu Z, Duan C, Zhang Y, Wu X, Wu H (2023). Role of human papillomavirus and associated viruses in bladder cancer: An updated review. J Med Virol.

[22] Ando T, Tai-Nagara I, Sugiura Y, Kusumoto D, Okabayashi K, Kido Y (2022). Tumor-specific interendothelial adhesion mediated by FLRT2 facilitates cancer aggressiveness. J Clin Invest.

[23] Jiang P, Ning J, Yu W, Rao T, Ruan Y, Cheng F (2024). FLRT2 suppresses bladder cancer progression through inducing ferroptosis. J Cell Mol Med.

[24] Zhang J, Zhang X, Su J, Zhang J, Liu S, Han L (2023). Identification and validation of a novel HOX-related classifier signature for predicting prognosis and immune microenvironment in pediatric gliomas. Front Cell Dev Biol.

[25] Long Z, Sun C, Tang M, Wang Y, Ma J, Yu J (2022). Single-cell multiomics analysis reveals regulatory programs in clear cell renal cell carcinoma. Cell Discovery.

[26] Revilla G, Ruiz-Auladell L, Vallverdú NF, Santamaría P, Moral A, Pérez JI (2023). Low-Density Lipoprotein Receptor Is a Key Driver of Aggressiveness in Thyroid Tumor Cells. Int J Mol Sci.

[27] Wang BY, Chang YY, Shiu LY, Lee YJ, Lin YW, Hsu YS (2023). An integrated analysis of dysregulated SCD1 in human cancers and functional verification of miR-181a-5p/SCD1 axis in esophageal squamous cell carcinoma. Comput Struct Biotechnol J.

[28] Guo Z, Huo X, Li X, Jiang C, Xue L (2023). Advances in regulation and function of stearoyl-CoA desaturase 1 in cancer, from bench to bed. Sci China Life Sci.

[29] Creighton CJ, Zhang F, Zhang Y, Castro P, Hu R, Islam M (2023). Comparative and integrative analysis of transcriptomic and epigenomic-wide DNA methylation changes in African American prostate cancer. Ciba F Symp.

[30] Lapcik P, Sulc P, Janacova L, Jilkova K, Potesil D, Bouchalova P (2023). Desmocollin-1 is associated with pro-metastatic phenotype of luminal A breast cancer cells and is modulated by parthenolide. Cell Mol Biol Lett.

[31] Faktor J, Knopfova L, Lapcik P, Janacova L, Paralova V, Bouchalova P (2019). Proteomics Identification and Validation of Desmocollin-1 and Catechol-O-Methyltransferase as Proteins Associated with Breast Cancer Cell Migration and Metastasis. Proteomics.

[32] Cañellas-Socias A, Cortina C, Hernando-Momblona X, Palomo-Ponce S, Mulholland EJ, Turon G (2022). Metastatic recurrence in colorectal cancer arises from residual EMP1+ cells. Nature.

[33] Zhou T, Chen H, Wang Y, Wen S, Dao P, Chen M (2023). Key Molecules in Bladder Cancer Affect Patient Prognosis and Immunotherapy Efficacy: Further Exploration for CNTN1 and EMP1. JCO Precis Oncol.

[34] Zheng Q, Luo Z, Xu M, Ye S, Lei Y, Xi Y (2023). HMGA1 and FOXM1 Cooperate to Promote G2/M Cell Cycle Progression in Cancer Cells. Life.

[35] Chia L, Wang B, Kim JH, Luo LZ, Shuai S, Herrera I (2023). HMGA1 induces FGF19 to drive pancreatic carcinogenesis and stroma formation. J Clin Invest.

[36] Chang X, Liu J, Yang Q, Gao Y, Ding X, Zhao J (2023). Targeting HMGA1 contributes to immunotherapy in aggressive breast cancer while suppressing EMT. Biochem Pharmacol.

[37] Takai M, Takamoto M, Amano Y, Yamamoto M, Hara K, Yashiro N (2023). Induction of lysophosphatidic acid (LPA) receptor-mediated signaling regulates cell motility and survival to anticancer drugs in cancer cells treated with hydrogen peroxide. Adv Biol Regul.

[38] Konen JM, Rodriguez BL, Wu H, Fradette JJ, Gibson L, Diao L (2023). Autotaxin suppresses cytotoxic T cells via LPAR5 to promote anti-PD-1 resistance in non-small cell lung cancer. J Clin Invest.

[39] Luo Z, Dong X, Yu J, Xia Y, Berry KP, Rao R (2021). Genomic and Transcriptomic Analyses Reveals ZNF124 as a Critical Regulator in Highly Aggressive Medulloblastomas. Front Cell Dev Biol.

[40] Meškytė EM, Pezzè L, Bartolomei L, Forcato M, Bocci IA, Bertalot G (2023). ETV7 reduces inflammatory responses in breast cancer cells by repressing the TNFR1/NF-κB axis. Cell Death Dis.

[41] Harwood FC, Klein Geltink RI, O'Hara BP, Cardone M, Janke L, Finkelstein D (2018). ETV7 is an essential component of a rapamycin-insensitive mTOR complex in cancer. Sci Adv.

[42] Jiang K, Wu L, Yin X, Tang Q, Yin J, Zhou Z (2022). Prognostic implications of necroptosis-related long noncoding RNA signatures in muscle-invasive bladder cancer. Front Genet.

[43] Jennis M, Kung CP, Basu S, Budina-Kolomets A, Leu JIJ, Khaku S (2016). An African-specific polymorphism in the TP53 gene impairs p53 tumor suppressor function in a mouse model. Genes Dev.

[44] Larsson SC, Lee WH, Burgess S, Allara E (2021). Plasma Cortisol and Risk of Atrial Fibrillation: A Mendelian Randomization Study. J Clin Endocrinol Metab.

[45] Jiang Z, Elsarrag SZ, Duan Q, LaGory EL, Wang Z, Alexanian M (2022). KLF15 cistromes reveal a hepatocyte pathway governing plasma corticosteroid transport and systemic inflammation. Sci Adv.

[46] Desgrosellier JS, Lesperance J, Seguin L, Gozo M, Kato S, Franovic A (2014). Integrin αvβ3 drives slug activation and stemness in the pregnant and neoplastic mammary gland. Dev Cell.

[47] Li D, Zhang Y, Zhang H, Zhan C, Li X, Ba T (2018). CADM2, as a new target of miR-10b, promotes tumor metastasis through FAK/AKT pathway in hepatocellular carcinoma. J Exp Clin Canc Res.

[48] Yang TY, Wu ML, Chang CI, Liu CI, Cheng TC, Wu YJ (2018). Bornyl cis-4-Hydroxycinnamate Suppresses Cell Metastasis of Melanoma through FAK/PI3K/Akt/mTOR and MAPK Signaling Pathways and Inhibition of the Epithelial-to-Mesenchymal Transition. Int J Mol Sci.

[49] Lawrence MS, Stojanov P, Mermel CH, Robinson JT, Garraway LA, Golub TR (2014). Discovery and saturation analysis of cancer genes across 21 tumour types. Nature.

[50] De Santis M, Sala V, Martini M, Ferrero G, Hirsch E (2017). PI3K Signaling in Tissue Hyper-Proliferation: From Overgrowth Syndromes to Kidney Cysts. Cancers.

[51] Ye B, Jiang LL, Xu HT, Zhou DW, Li ZS (2012). Expression of PI3K/AKT pathway in gastric cancer and its blockade suppresses tumor growth and metastasis. Int J Immunopath Ph.

[52] Bakin AV, Tomlinson AK, Bhowmick NA, Moses HL, Arteaga CL (2000). Phosphatidylinositol 3-kinase function is required for transforming growth factor beta-mediated epithelial to mesenchymal transition and cell migration. J Biol Chem.

[53] Clause KC, Barker TH (2013). Extracellular matrix signaling in morphogenesis and repair. Curr Opin Biotechnol.

[54] Riegler J, Labyed Y, Rosenzweig S, Javinal V, Castiglioni A, Dominguez CX (2018). Tumor Elastography and Its Association with Collagen and the Tumor Microenvironment. Clin Cancer Res.

[55] Brooks M, Mo Q, Krasnow R, Ho PL, Lee YC, Xiao J (2016). Positive association of collagen type I with non-muscle invasive bladder cancer progression. Oncotarget.

[56] Genard G, Wera AC, Huart C, Le Calve B, Penninckx S, Fattaccioli A (2018). Proton irradiation orchestrates macrophage reprogramming through NFκB signaling. Cell Death Dis.

[57] Sun M, Zeng H, Jin K, Liu Z, Hu B, Liu C (2022). Infiltration and Polarization of Tumor-associated Macrophages Predict Prognosis and Therapeutic Benefit in Muscle-Invasive Bladder Cancer. Cancer Immunol Immunother.

[58] Buckley MT, Yoon J, Yee H, Chiriboga L, Liebes L, Ara G (2007). The histone deacetylase inhibitor belinostat (PXD101) suppresses bladder cancer cell growth *in vitro* and *in vivo*. J Transl Med.

[59] Klein FG, Granier C, Zhao Y, Pan Q, Tong Z, Gschwend JE (2021). Combination of Talazoparib and Palbociclib as a Potent Treatment Strategy in Bladder Cancer. J Pers Med.

[60] Zhao Z, Liu S, Sun R, Zhu W, Zhang Y, Liu T (2023). The combination of oxaliplatin and anti-PD-1 inhibitor promotes immune cells infiltration and enhances anti-tumor effect of PD-1 blockade in bladder cancer. Front Immunol.

[61] Camargo JA, Passos GR, Ferrari KL, Billis A, Saad MJA, Reis LO (2018). Intravesical Immunomodulatory Imiquimod Enhances Bacillus Calmette-Guérin Downregulation of Nonmuscle-invasive Bladder Cancer. Clin Genitourin Cancer.

[62] Polastro L, Aftimos PG, Awada A (2014). Eribulin mesylate in the management of metastatic breast cancer and other solid cancers: a drug review. Expert Rev Anticancer Ther.

[63] Joshi M, Holder SL, Zhu J, Zheng H, Komanduri S, Warrick J (2022). Avelumab in Combination with Eribulin Mesylate in Metastatic Urothelial Carcinoma: BTCRC GU-051, a Phase 1b Study. Eur Urol Focus.

