# Supplementary material for: HPV-Associated Gene Signatures in Bladder Cancer: A Comprehensive Prognostic Model and its Implications in Immunotherapy
Source: Int J Med Sci. 2025 Jan 1;22(1):140–57. doi: 10.7150/ijms.98334 (PMC11659835; doi:10.7150/ijms.98334)
Supplement: Supplementary file 1 — Supplementary figures and tables. [file ijmsv22p0140s1.pdf]

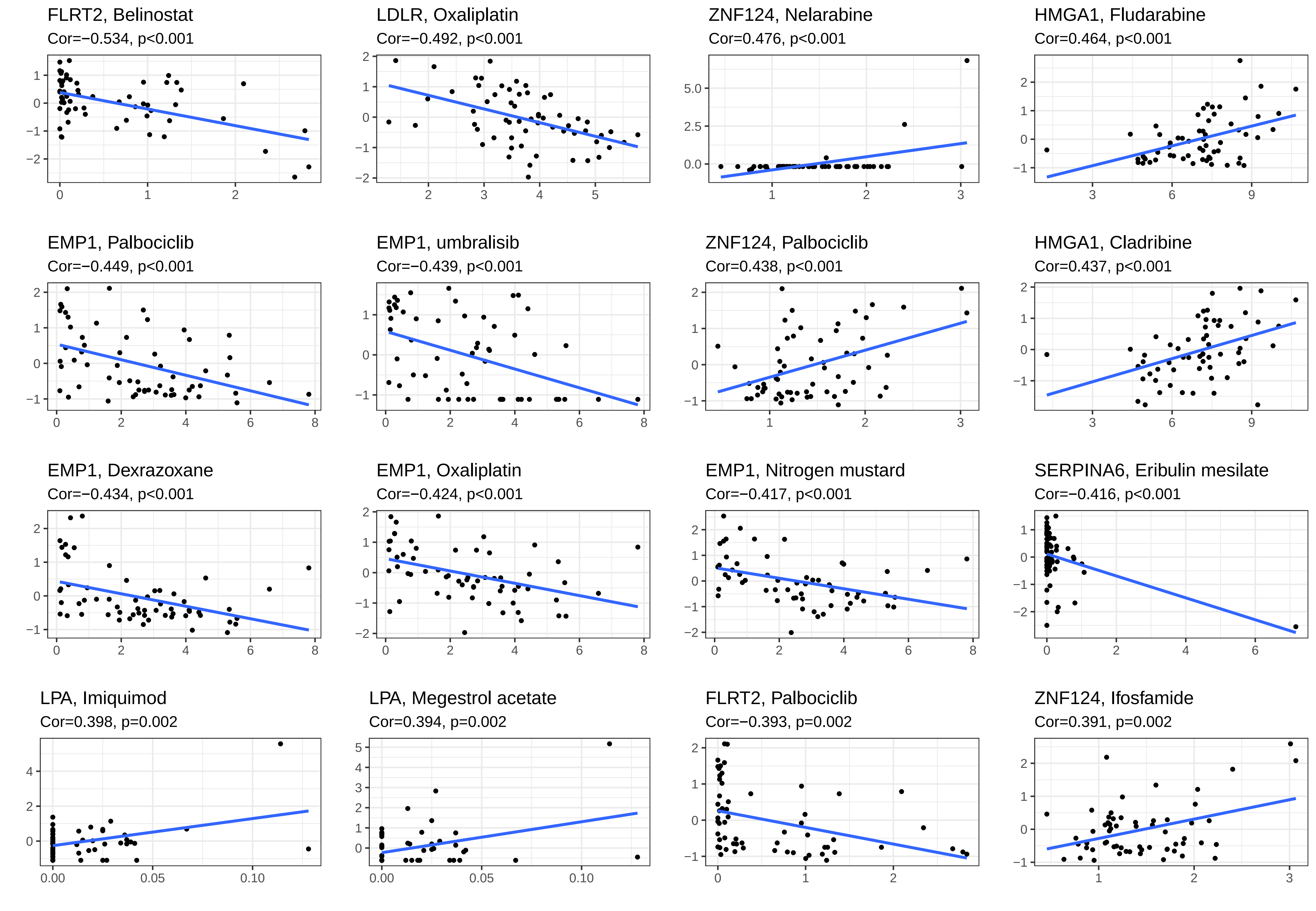
FLRT2, Belinostat
Cor=−0.534, p<0.001
LDLR, Oxaliplatin
Cor=−0.492, p<0.001
ZNF124, Nelarabine
Cor=0.476, p<0.001
HMGA1, Fludarabine
Cor=0.464, p<0.001
EMP1, Palbociclib
Cor=−0.449, p<0.001
EMP1, umbralisib
Cor=−0.439, p<0.001
ZNF124, Palbociclib
Cor=0.438, p<0.001
HMGA1, Cladribine
Cor=0.437, p<0.001
EMP1, Dexrazoxane
Cor=−0.434, p<0.001
EMP1, Oxaliplatin
Cor=−0.424, p<0.001
EMP1, Nitrogen mustard
Cor=−0.417, p<0.001
SERPINA6, Eribulin mesilate
Cor=−0.416, p<0.001
LPA, Imiquimod
Cor=0.398, p=0.002
LPA, Megestrol acetate
Cor=0.394, p=0.002
FLRT2, Palbociclib
Cor=−0.393, p=0.002
ZNF124, Ifosfamide
Cor=0.391, p=0.002

Gene name	Primer type	Primer sequence
HMGA1	Forward primer	GCTGGTAGGGAGTCAGAAGGA
	Reverse primer	TGGTGGTTTTCCGGGTCTTG
SCD	Forward primer	TCTAGCTCCTATACCACCACCA
	Reverse primer	TCGTCTCCAACTTATCTCCTCC
SCO2	Forward primer	ACAGGCTCTCTCAGCTCAAG
	Reverse primer	CAGGGCTTCTGTTCGCTTTTG
LDLR	Forward primer	TCTGCAACATGGCTAGAGACT
	Reverse primer	TCCAAGCATTCGTTGGTCCC
FLRT2	Forward primer	CGCTGCGACAGGAACTTTG
	Reverse primer	TGGAGGTAGAGTACGGTTACG
HOXC5	Forward primer	AGAGCCCCAATATCCCTGC
	Reverse primer	CGGTGGGAAAGTGATGCTT
GRM7	Forward primer	ACAGGCTCTCTCAGCTCAAG
	Reverse primer	CAGGGCTTCTGTTCGCTTTTG
EMP1	Forward primer	TCTGCAACATGGCTAGAGACT
	Reverse primer	TCCAAGCATTCGTTGGTCCC
LPA	Forward primer	TCCGAACAAGCACCGACTG
	Reverse primer	GGTCCGACTATGCGAGTGT
SERPINA6	Forward primer	GTGAACATGAGTAACCATCACCG
	Reverse primer	CCTGGTGGATCTCAGTCTCAG
ZNF124	Forward primer	TGTGGGAAAGCCTTAGGTTTTT
	Reverse primer	ACATGGATAGGGTTCTTCACCA
ETV7	Forward primer	CTGCTGTGGGATTACGTGTATC
	Reverse primer	GTTCTTGTGATTTCCCCAGAGTC
DSC1	Forward primer	AGGCTGAAACACTTGTAGGCA
	Reverse primer	GTTGTTCCCGTCTCTGACCAT

Supplementary Table.The primers used for RT-qPCR
